# Hybrid Machine-Learning-Based Prediction Model for the Peak Dilation Angle of Rock Discontinuities

**DOI:** 10.3390/ma16196387

**Published:** 2023-09-24

**Authors:** Shijie Xie, Rubing Yao, Yatao Yan, Hang Lin, Peilei Zhang, Yifan Chen

**Affiliations:** 1 School of Civil Engineering, Southeast University, Nanjing 210096, China; 2 School of Resources and Safety Engineering, Central South University, Changsha 410083, China18570770169@163.com (Y.C.); 3 School of Qilu Transportation, Shandong University, Jinan 250061, China

**Keywords:** rock, peak dilation angle, machine learning, support vector regression, mechanical properties

## Abstract

The peak dilation angle is an important mechanical feature of rock discontinuities, which is significant in assessing the mechanical behaviour of rock masses. Previous studies have shown that the efficiency and accuracy of traditional experimental methods and analytical models in determining the shear dilation angle are not completely satisfactory. Machine learning methods are popular due to their efficient prediction of outcomes for multiple influencing factors. In this paper, a novel hybrid machine learning model is proposed for predicting the peak dilation angle. The model incorporates support vector regression (SVR) techniques as the primary prediction tools, augmented with the grid search optimization algorithm to enhance prediction performance and optimize hyperparameters. The proposed model was employed on eighty-nine datasets with six input variables encompassing morphology and mechanical property parameters. Comparative analysis is conducted between the proposed model, the original SVR model, and existing analytical models. The results show that the proposed model surpasses both the original SVR model and analytical models, with a coefficient of determination (R^2^) of 0.917 and a mean absolute percentage error (MAPE) of 4.5%. Additionally, the study also reveals that normal stress is the most influential mechanical property parameter affecting the peak dilation angle. Consequently, the proposed model was shown to be effective in predicting the peak dilation angle of rock discontinuities.

## 1. Introduction

**Figure 1:**
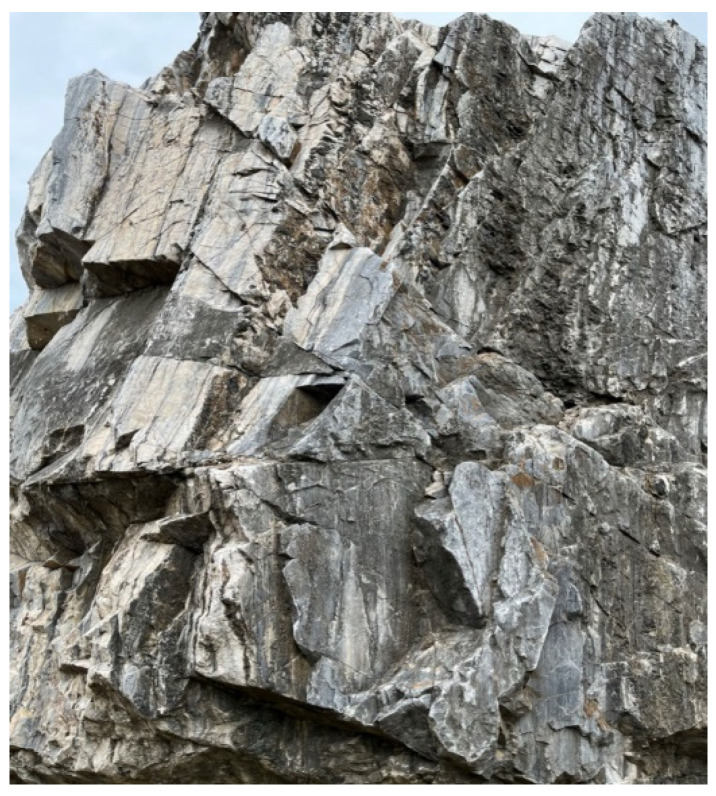
Photograph of rock outcrops in China.

The forecasting and control of the mechanical behaviour of rock masses is an important factor regarding the safety of engineering structures [1,2,3,4,5]. The design of structures such as tunnels, embankments, mine openings, and underground chambers relies on accurate and reliable estimates of compressive strength, tensile strength, hydraulic mechanics, internal damage characteristics, and shear strength of rock masses [6,7,8,9,10,11]. It is generally accepted that rock masses are often cut into intact rock pieces by rock discontinuities at different scales, as shown in Figure 1. These rock discontinuities include fractures, joints, bedding planes, weak intercalations, shear planes, etc. [12,13]. Due to the shear strength of rock discontinuities closely related to rock engineering disasters, such as rock slope failure, fault-slip burst, and collapse accidents in tunnels [14,15,16], it has attracted the attention of researchers [17,18,19,20,21].

The Mohr–Coulomb law is widely used to characterise shear behaviours of rock discontinuities in the existing shear strength models, which incorporates the internal friction angle comprising the basic friction angle and the peak dilation angle [22,23]. At the same time, there is an excellent modern-day geotechnical software, FLAC3D5.0, in which this law is taken as a basis and allows for predicting the behaviour of rock masses in different conditions [24,25]. The peak dilation angle reflects the comprehensive effect of the joint morphology on the shear strength [26,27]. Generally speaking, the peak dilation angle is defined as the instantaneous inclination of the shear path at the shear strength with respect to the mean plane [28]. In addition, the peak dilation angle is also the most commonly used parameter in numerical calculations to study the nonlinear shear dilation behaviour of rock materials and to simulate surrounding rock deformation [29,30,31,32,33].

The dilation is also inherent to failure in specimens starting from intact material, and it is a fundamental parameter for models with softening or hardening behaviour, also modified by the average stress level acting along the stress path. Currently, numerous experimental studies [34,35] on the peak dilation angle have been carried out. Moreover, theoretical analysis and many empirical models [36,37] were established based on the concept of maximum dilation angle at zero normal stress. These models have some shortcomings. For example, Xia et al. [38] proposed a new empirical model by tensile joint replicas satisfying new peak dilation angle boundary conditions under zero and critical state normal stresses. However, as the normal stress increases, the peak dilation angle predicted by the Xia et al. [38] model is half of the initial dilatation angle, which is inconsistent with the actual behaviour [39]. For this, Yang et al. [39] established a new empirical model based on the shear test results of granite joints and sandstone joints. Ban et al. [40] also took into account the real contact asperity distribution and proposed a semi-empirical model. Additionally, there are many empirical models for predicting the peak dilation angle and shear strength of rock discontinuities, as listed in Table 1. These models provide a valuable basis for understanding and predicting the peak dilation angle. However, the generality of these models has not been well-addressed and some model parameters lack clear physical meaning. From an engineering practicality point of view, an ideal model should be able to accurately assess the peak dilation angle in a time-saving, labour-saving, and cost-effective way.

Rock materials exhibit complex behaviours and a high level of uncertainty under laboratory testing [49,50,51,52,53,54]. Machine learning (ML) techniques have been developed and used by an increasing number of researchers in the last several decades [55,56,57,58,59,60,61,62]. Compared with traditional test methods and empirical models, ML can effectively find implicit relationships between variables and well handle nonlinear problems [63,64]. The support vector regression (SVR) algorithm presents high accuracy and efficiency in modelling the nonlinear association between input variables and outputs, and it has been widely used in rock mechanics modelling in recent years [65]. For example, Huang et al. [66] used the joint roughness coefficient (JRC), uniaxial compressive strength, normal stress, and basic friction angle as the input variables of the SVR model to intelligently predict the shear strength. Under the framework of SVR, Babanouri and Fattahi [67] proposed a new shear constitutive model of rock discontinuity. Ceryan et al. [68] developed an SVR model to predict the elastic modulus of rock materials with different degrees of weathering. Recently, Xu et al. [69] used SVR to study multiple geomechanical properties of rock materials. In conclusion, SVR exhibits several distinct advantages when tackling challenges involving high-dimensional and nonlinear recognition problems. 

It can be noted that the peak dilation angle model of rock discontinuities is a very topical issue. Therefore, the purpose of this study is to provide an efficient method for predicting the peak dilation angle of rock discontinuities and to achieve this. The grid search optimization algorithm (GS) is introduced to improve the effect of the SVR, and a hybrid machine learning model, the GS-SVR model, is proposed. In addition, to show the development process of the proposed model, detailed analysis and model performance are also presented. Finally, the limitations and future development progress of the proposed model are outlined.

## 2. Methodological Background

### 2.1. SVR

**Figure 2:**
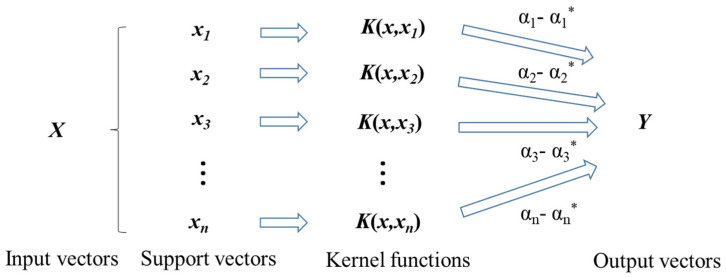
Schematic diagram of the SVR.

As a typical kernel-based ML algorithm, SVR is a promotion of support vector machine (SVM). It also follows the function approximation algorithm of SVM and solves the multivariate nonlinear regression estimation problem by introducing an alternative loss function [70]. As a supervised learning method based on the principle of structural risk minimization, SVR has good generalization ability in solving small-sample, nonlinear, and high-dimensional problems [71]. Because it is a convex quadratic optimization technique, it can always achieve the global optimal solution [72]. Figure 2 displays a schematic diagram of the SVR employed in this paper. SVR uses nonlinear mapping to translate the input vector X into a space with higher dimensions. More details about SVR and its application can be found in other milestone papers [73,74,75]. In this work, SVR is chosen as the regression tool to predict the peak dilation angle because of its high generalization performance. It is worth mentioning that the relationship between peak dilation angle and underlying variables is nonlinear, high-dimensional, and the training data are generally not large. That circumstance is particularly suitable for SVR.

For a certain set of training data {(*x*_1_, *y*_1_), (*x*_2_, *y*_2_), …(*x_n_*, *y_n_*)}, the aim is to seek an optimal function *f*(*x*) that has at most ε deviation from the target values *y_tar_* for all the training data. The optimal function *f*(*x*) that has the most ε deviation from the target value in ε-SVR can be written as Equation (1):

(1) $$f\left(x\right)=\sum_{n=1}^{N}\omega \varphi_{n}\left(x\right)+b$$

where *ω* is the weight vector, *b* is the model error, *N* represents the total number of training data, *φ_n_*(*x*) denotes a nonlinear mapping function.

Subsequently, the overall optimization is optimally transformed into Equation (2).

(2) $$Minimize\frac{1}{2}{\left\|\omega \right\|}^{2}$$

where the Euclidean norm $\frac{1}{2}{\left\|\omega \right\|}^{2}$ is $\frac{1}{2}\omega^{T}\omega$.

The constraints of Equation (2) are shown below:

(3) $$\{\begin{array}{c} y_{i}-\omega x_{i}-b\leq \varepsilon \\ \omega^{T}x_{i}+b-y_{i}\leq \varepsilon \end{array}$$

By introducing two slack variables *ξ_i_* and *ξ_i_^∗^* (*i* = 1, 2…, *n*) into Equation (3) representing the separation between the actual values and corresponding boundary values of ε-deviation. Further, the *w* and *b* can be determined by minimizing the following optimization function

(4) $$\begin{array}{cc} Minimize & \frac{1}{2}{\left\|\omega \right\|}^{2}+c\sum_{i}^{n}\left(\xi_{i}+\xi_{i}^{*}\right)\\ Subjectto & \{\begin{array}{c} y_{i}-\omega^{T}\varphi \left(x_{i}\right)-b\leq \varepsilon +\xi_{i}\\ \omega^{T}\varphi \left(x_{i}\right)+b-y_{i}\leq \varepsilon +\xi_{i}^{*}\\ \xi_{i},\xi_{i}^{*}\geq 0 \end{array} \end{array}$$

where *c* is the regularization or penalty parameter that is greater than zero.

The $\frac{1}{2}{\left\|\omega \right\|}^{2}$ term denotes the structure risk and the $c\sum_{i}^{n}\left(\xi_{i}+\xi_{i}^{*}\right)$ second term represents the empirical risk. Equation (4) is a constrained optimization problem that can be transformed in the form of a Lagrange function *L*(*α*, α^*^) by sequential minimal optimization algorithm in a dual form:

(5) $$\begin{array}{cc} Maximize & L\left(\alpha ,\alpha^{*}\right)=-\frac{1}{2}\sum_{i=1}^{n}\sum_{j=1}^{n}\left(\alpha_{i}-\alpha_{i}{}^{*}\right)\left(\alpha_{j}-\alpha_{j}{}^{*}\right)K\left(x_{i},x_{j}\right)+\sum_{i=1}^{n}y_{i}\left(\alpha_{i}-\alpha_{i}{}^{*}\right)-\varepsilon \sum_{i=1}^{n}y_{i}\left(\alpha_{i}+\alpha_{i}{}^{*}\right)\\ Subjectto & \{\begin{array}{c} \sum_{i=1}^{n}\left(\alpha_{i}-\alpha_{i}{}^{*}\right)=0\\ 0\leq \alpha_{i}\leq c\\ 0\leq \alpha_{i}{}^{*}\leq c \end{array} \end{array}$$

where *α_i_* and α_i_^*^ are the Lagrangian multipliers, *K*(*x_i_*,*x_j_*) = *φ*(*x_i_*)*φ*(*x_j_*) is the kernel function that yields the inner product in a higher-dimensional feature space.

By using *K*(*x_i_*,*x_j_*), one can directly transform the data into a higher-dimensional feature without calculating the explicit map *φ*(*x*). In this paper, the radial basis function kernel function (RBF) is employed because of its high generalization performance.

(6) $$K\left(x_{i},x_{j}\right)=e^{{\left(-g\left\|x_{i}-x_{j}\right\|\right)}^{2}}$$

where *g* denotes the kernel parameter, $\left\|x_{i}-x_{j}\right\|$ is the Euclidean distance.

The nonlinear regression function can be expressed as follows after taking the Lagrangian and optimum conditions into account:

(7) $$f\left(x\right)=\sum_{n=1}^{N}\left(\alpha_{i}-\alpha_{i}{}^{*}\right)K\left(x_{i},x_{j}\right)+b$$

### 2.2. GS Optimization

In order to achieve accurate prediction, an important issue to be concerned with in implementing the SVR model is the tuning of hyperparameters (e.g., penalty parameter *c* and width parameter *g*). The trade-off between model complexity and training error is controlled by *c*, while the complexity of the solution is determined by *g*. The tuning process is generally completed through optimization algorithms.

**Figure 3:**
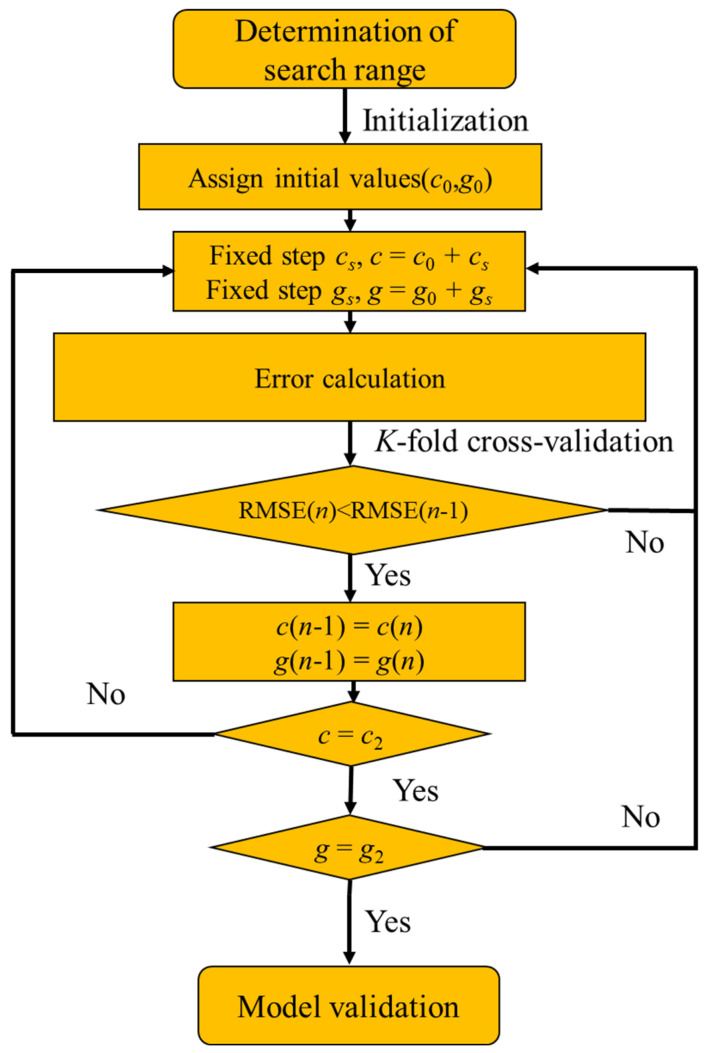
Computation flowchart of grid search method.

As a classical parameter optimization method, the grid search (GS) method is proved to be an efficient optimization method with ideal convergence speed and success rate [76]. It is a method of optimizing the performance of a model by traversing a given combination of parameters, by testing all combinations of a given parameter and finding the most suitable combination. The specific optimization process is shown in Figure 3. The evaluation metrics, such as root mean square error (RMSE), coefficient of determination (R^2^), and mean squared error (MSE), are obtained using *K*-fold cross-validation for all hyperparameter combinations of the selected grid nodes. The best combination of *c* and *g*, which resulted in the best performance of the evaluation metrics, was selected for subsequent model validation.

## 3. Data Pre-Processing

A dataset with reliable experimental results and wide distribution is a prerequisite for the successful application of ML modelling [77,78]. Based on the literature review of the existing research method [39,40], six parameters, including normal stress, basic friction angle, three-dimensional roughness parameters, and uniaxial compressive strength, were selected as the input variables of the proposed model.

The results of joint shear tests available in the literature are compiled. The dataset consists of 89 shear test results from various experimental results collected by the authors. These test results cover common joint types, such as cement mortar [40], granite [39,79], sandstone [38,39,80], marble [79], and limestone [79], and the projected lengths of these rock discontinuities ranged from 140 to 300 mm. More information on sample preparation procedures can be found in the corresponding literature. Detailed information on rock type, sample size, normal stress (*σ_n_*), mechanical properties (uniaxial compressive strength *σ_c_* and basic friction angle *φ_b_*), three-dimensional roughness parameters (*A*_0_, *C*, θ_max_^*^), and measured peak dilation angle (*i_p_*) collected in the dataset are shown in the Supplemental Files. A detailed statistical description of the input variables and output variable is shown in Table 2. As shown, there is an evident difference in the data distribution (e.g., data scope, magnitude difference) for variables. Therefore, in order to speed up the computational efficiency and convergence of ML, all inputs and output need to be normalized to (0,1) range according to their maximum and minimum values. The normalization formula is shown in Equation (8) as follows:

(8) $$\{\begin{array}{c} x_{i}’=\frac{x_{i}-x_{\min}}{x_{\max}-x_{\min}}\\ y_{i}’=\frac{y_{i}-y_{\min}}{y_{\max}-y_{\min}} \end{array}$$

where *x_i_*^’^ and *y_i_*^’^ represent normalized input and output values of the *i*-th sample; *x_i_* and *y_i_* represent experimental input and output values of the *i*-th sample; *x_min_*, *x_max_*, *y_min_*, *y_max_* represent corresponding minimum and maximum values.

**Figure 4:**
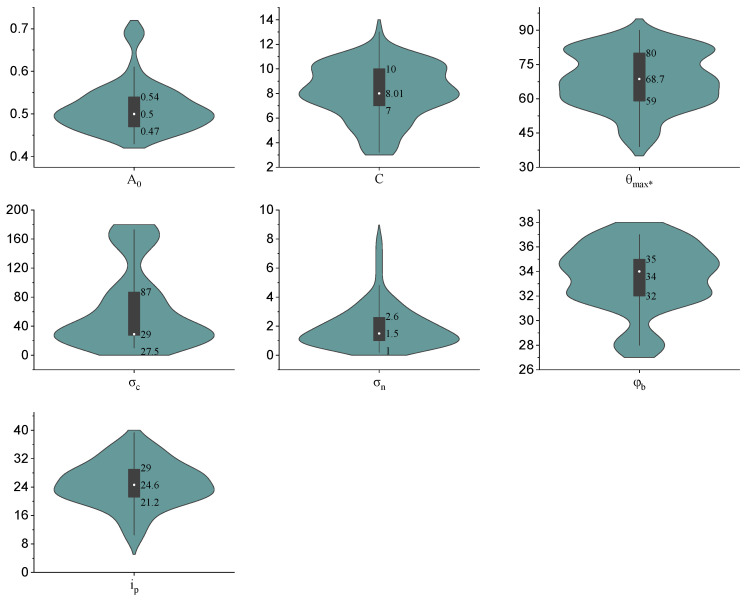
Violin plots of variables used in the database.

**Figure 5:**
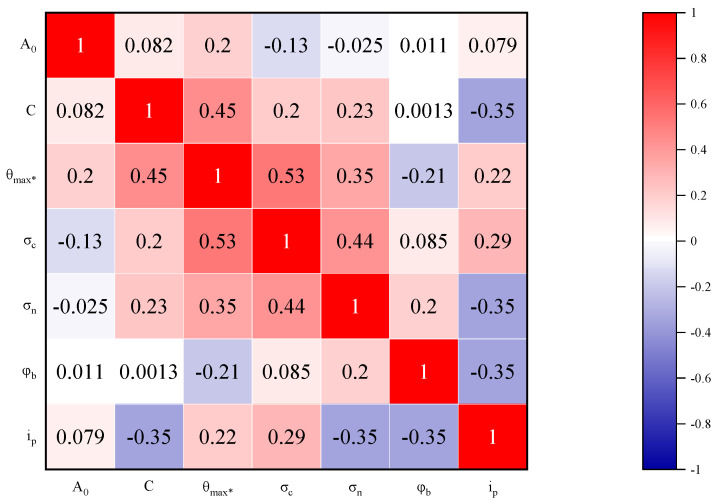
Correlation coefficients plot between input and output variables.

The distribution characteristics of the dataset are visualized by means of a violin plot, as shown in Figure 4. It combines the features of a kernel density plot and a box plot while showing the first quartile, median, and third quartile of the dataset. A matrix analysis was plotted to show the correlation coefficients between the variables, with negative numbers representing negative correlations. It is easy to see from Figure 5 that all the correlation coefficients are less than 0.53, which indicates that these input variables are independent of each other and do not cause multicollinearity problems. Moreover, the correlation coefficients between the input and output variables are relatively low (all values are less than 0.35 in absolute value), which indicates that the relationship between the peak dilation angle and these inputs is not a simple multivariate linear relationship but a complex nonlinear mapping relationship. In other words, it is difficult to establish an explicit equation between the peak dilation angle and the inputs. This is the reason why machine learning methods are used to predict the peak dilation angle in this paper.

## 4. Results and Comparisons

### 4.1. Hyperparameters Tuning Process

**Figure 6:**
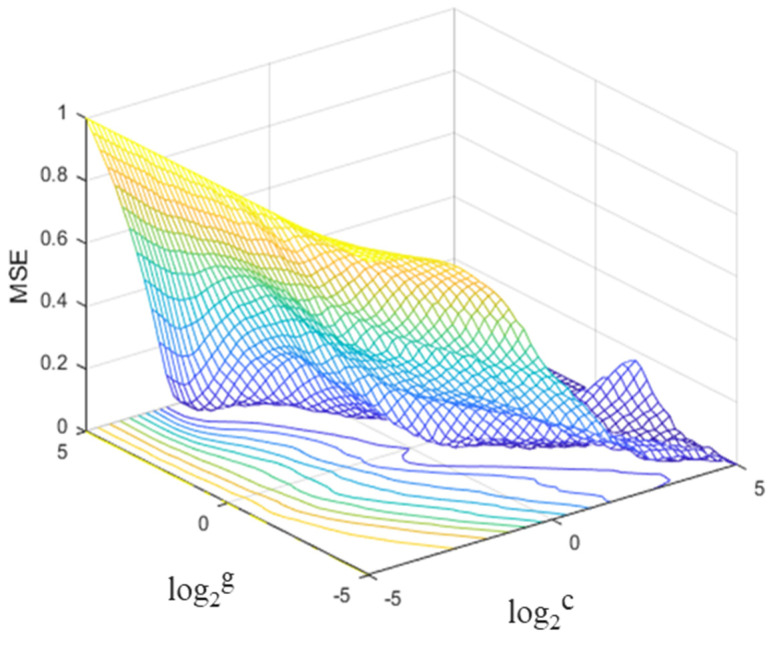

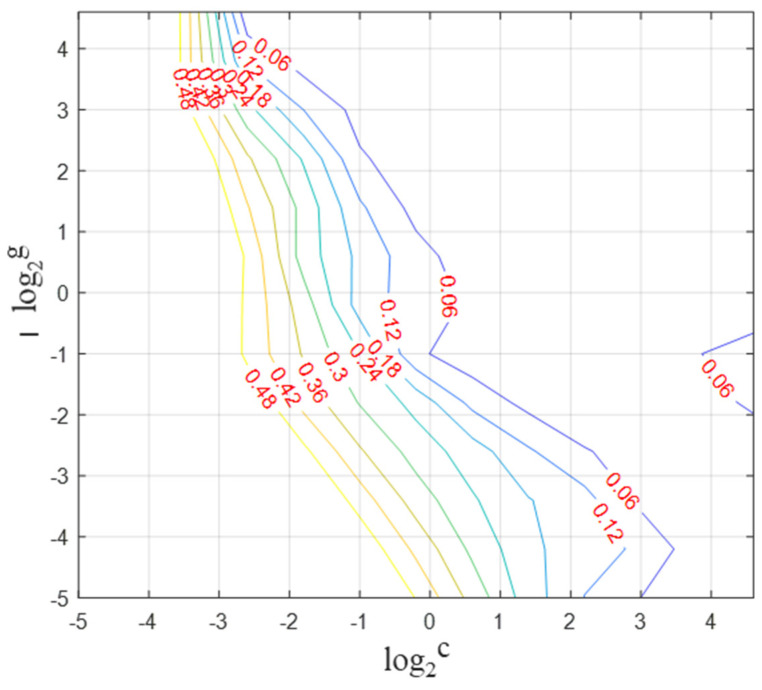
Solving hyperparameters using GS method.

The hyperparameters *c* and *g* have a significant effect on the performance of the prediction model. The grid is divided into a range of coordinates and according to a specified step, and all grids are traversed. The evaluation metrics (e.g., RMSE and MSE) are obtained by searching all combinations of parameters *c* and *g* for each selected grid node one by one using *K*-fold cross-validation. *K*-fold cross-validation is a statistical technique that can successfully remove the training bias brought on by sampling irrationality [73]. Subsequently, the search range and step are then adjusted according to the values of the evaluation metrics, and the best combination of *c* and *g* is the one that provides the best performance of the model cross-validation metrics. As shown in Figure 6, the search range for *c* and *g* was set to (2^−5^,2^5^) with a step of 2^0.2^. All grids were traversed and all combinations of parameters *c* and *g* were searched for each selected grid node one by one. The best model was determined with the lowest value of MSE using 5-fold cross-validation. Optimal solutions for the parameters in the search range are obtained in the optimal choice of parameters *c* and *g*. 

**Figure 7:**
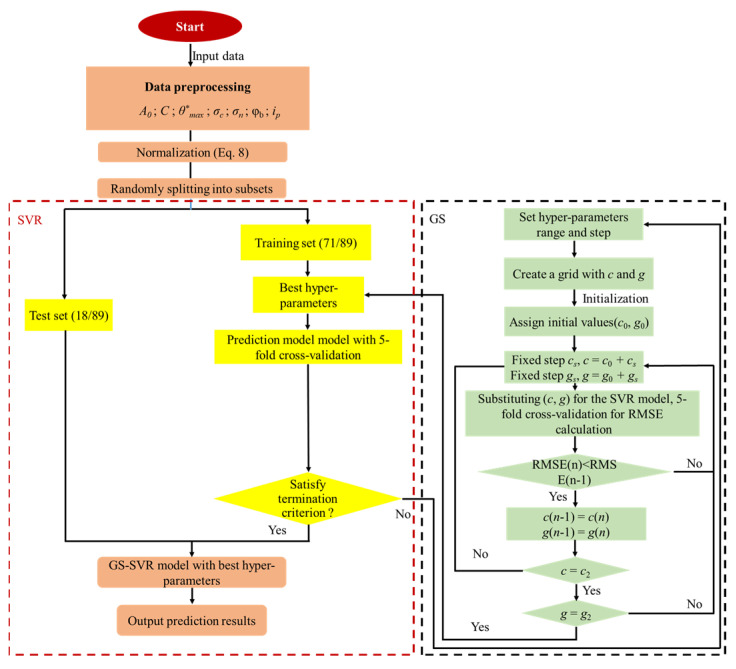
Prediction process of the proposed ML-based model.

After obtaining the optimal combination of hyperparameters, the framework of the GS-SVR model for estimating the peak dilation angle is shown in Figure 7. In machine learning, a training set is typically used to build the model and verify the model’s ability to predict new data on an independent test set [81]. Therefore, the original dataset is randomly divided into two subsets after the dataset normalization: the training set and the test set. Through optimization analysis, 80% of the entire dataset was included in the training set and the remaining 20% was included in the test set.

### 4.2. Performance of GS-SVR Model

The coefficient of determination (*R*^2^), adjusted *R*^2^ (Adj. *R*^2^), root mean square error (RMSE), and mean absolute percentage error (MAPE) have been widely used for the performance evaluation of ML. These four evaluation indices are used to characterize the relationship between the predicted and test values of the peak dilation angle. *R*^2^ is a comprehensive metric to measure how strong the relationship is between the two variables. Adj. *R*^2^ represents the ability to accurately predict samples. The RMSE is a metric for how much actual values vary from the average of the estimated values. The MAPE measures the average relative error between the estimated and actual values. Generally, *R*^2^ (Adj. *R*^2^) values equal to 1 and RMSE (MAPE) values equal to 0 indicate the best prediction performance. The mathematical expressions for these four evaluation indices are listed below [82]:

(9) $$R^{2}=1-\frac{\sum_{i=1}^{N}{\left(y_{i}^{m}-y_{i}^{p}\right)}^{2}}{\sum_{i=1}^{N}{\left(y_{i}^{m}-\bar{y^{m}}\right)}^{2}}$$

(10) $$A\mathrm{dj}.R^{2}=1-\left[\left(1-R^{2}\right)\times \frac{N-1}{N-m-1}\right]$$

(11) $$MAPE=\frac{1}{N}\sum_{i=1}^{N}\left|\frac{y_{{}^{i}}^{m}-y_{{}^{i}}^{p}}{y_{{}^{i}}^{m}}\right|\times 100\%$$

(12) $$RMSE=\sqrt{\frac{1}{N}\sum_{i=1}^{N}{\left(y_{{}^{i}}^{m}-y_{{}^{i}}^{p}\right)}^{2}}$$

where *y_i_^m^* is the measured results; *y_i_^p^* is the predicted results; $\bar{y^{m}}$ is the average of *y_i_^m^*; *N* represents the number of samples; *m* represents the number of input variables.

**Figure 8:**
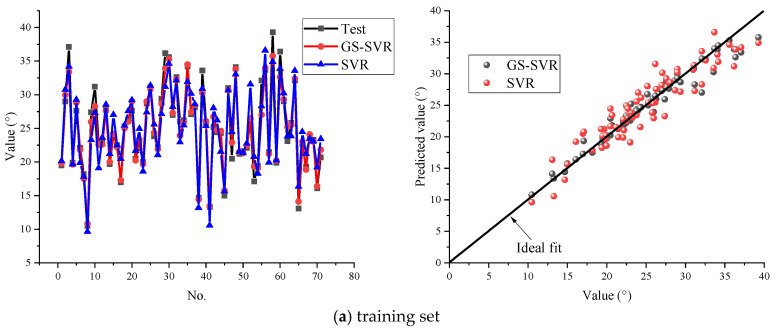

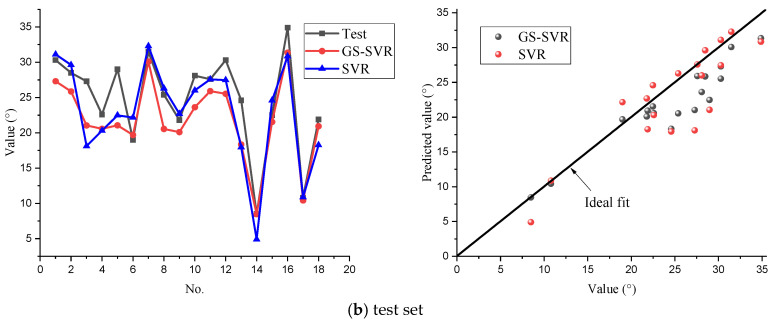
Performance of the model on the (**a**) training set; (**b**) test set.

In order to highlight the predictive performance of the proposed model, the original SVR model is also applied to the training set and test set. Figure 8 shows the prediction effect of the two models on the same training set and test set. The calculation of evaluation indices of the two models is shown in Table 3. It can be found that, compared with the SVR model, the GS-SVR model has a higher correlation coefficient (R^2^ and Adj. R^2^) and smaller error index (MAPE and RMSE). Figure 8b indicates that the GS-SVR model underestimates most of the test set and this is conducive to leaving some safety redundancy in engineering. Regardless of the training set or the test set, the predicted results of the GS-SVR model are distributed near the ideal fit line, and the predicted values are closer to the experimental results than the original SVR model. The values of evaluation indices shown in bold rows in Table 3 also indicate that the predicted values of the GS-SVR model are more consistent with the experimental values, and the predicted results are more accurate than those of the original SVR model.

### 4.3. Comparison with Existing Models

In order to verify the superiority of the new model, the proposed model is compared with six existing analytical models. The proposed model, Ban et al. (2023) model [22], Ban (2020) model [40], Grasselli (2001) model [79], Xia (2014) model [38], Yang (2016) model [39], and Tatone (2010) model [80] were, respectively, applied to this database, and the predictions of each model are shown in Table 4.

**Figure 9:**
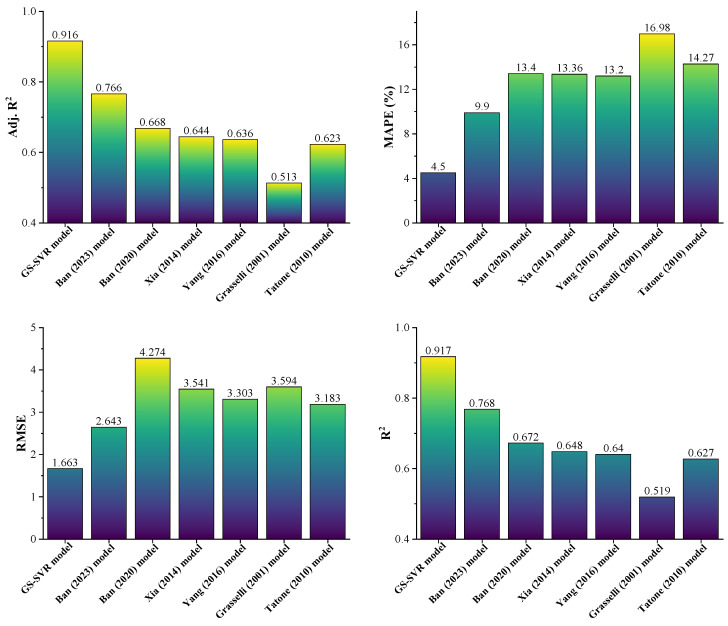
Performance comparison between the GS-SVR model and other models.

In order to make the estimation results more vivid, the estimation results and errors of each model are drawn, as shown in Figure 9. It can be seen from Figure 9 that the estimation performance of the GS-SVR model is significantly better than the other six models. The four evaluation indices shown in Figure 9 also indicate that the GS-SVR model outperformed the other six models in terms of predicting the peak dilation angle. That is, the lowest MAPE = 4.5% (RMSE = 1.663) and the highest R^2^ = 0.917 (Adj. R^2^ = 0.916) values for the dataset were obtained from the GS-SVR model. From this point of view, the GS-SVR model is easier and more robust than existing models. Interestingly, the evaluation indices for both training set (R^2^ = 0.92, RMSE = 1.138) and test set (R^2^ = 0.891, RMSE = 1.798) were similar to that obtained using the dataset (R^2^ = 0.917, RMSE = 1.663), which also suggests the GS-SVR model has similar accuracy in both fitting and prediction and has high generalizability.

## 5. Discussion

### 5.1. Relative Importance of Inputs

**Figure 10:**
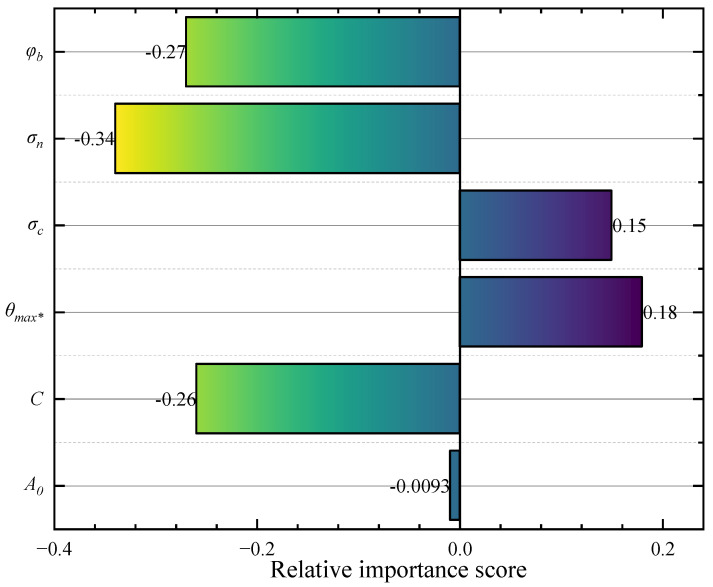
Relative importance score of input variables.

A sensitivity analysis of diverse input variables is carried out for a better understanding of the peak dilation angle. The method used for interpreting the relative importance of input variables is Kendall’s tau coefficient. Figure 10 demonstrates the obtained relative importance scores for each input variable. Note that each input variable contributes to the peak dilation angle, but with different levels of significance. It can be seen that the σ_n_ is the most sensitive variable for peak dilation angle. The influence of the *A*_0_ on the peak dilation angle is found to be the smallest among the input variables. The relative importance score of each input variable revealed important discoveries and indicated potential experimental studies of peak dilation angle. These findings might provide a more detailed understanding of the peak dilation angle and present potential experimental studies in the future. 

### 5.2. Contribution and Limitations

The primary advantage of this study is that a machine-learning-based model for predicting peak dilation angle is proposed. This method can provide a low-cost, time-saving, and non-destructive prediction of peak dilation angle for relevant geotechnical engineering, especially for projects with time and budget constraints.

Compared with the existing prediction models, the method has the following advantages: (1) the GS-SVR model does not require any mechanical testing after model training is completed; (2) the generalization capability of the GS-SVR model can be easily improved using large datasets, which may be better than the empirical equations established between the peak dilation angle and each influencing variable; (3) compared with the six analytical models to predict peak dilation angle, the advantages of ML techniques are strong data compatibility and model generalization. The accuracy of the GS-SVR model is the highest relative to the six analytical models.

There are still some shortcomings that need to be explored in the future. The scale effect is an important research topic in rock mechanics, and the effect of scale on shear mechanical behaviour of rock discontinuities is still unknown. In rock engineering design, the accurate understanding and mastering of the law of rock scale effect is related to the selection of rock mechanics parameters. How to extend the proposed model based on laboratory test results to the engineering scale is the next important research topic, and how to apply this model to industrialization is also an interesting direction. It might be necessary to create a graphics user interface (GUI). The omission of factors such as water content, shear displacement rate, and temperature is also a clear limitation of this study. In addition, as a data-driven approach, the predictive performance of the proposed model is severely affected by the quantity and quality of the Supporting Dataset. The method might be limited in some cases if there are information restrictions or not enough rock samples available. The final limitation is that the generalization capability of the proposed model on completely unknown test results (e.g., not included in this dataset) has not been fully investigated. 

## 6. Conclusions

This paper intends to provide an efficient method for predicting the peak dilation angle of rock discontinuities using a machine learning tool. The method is a hybrid GS-SVR model, which incorporates support vector regression (SVR) techniques and augments with the grid search optimization algorithm to improve prediction performance and optimize hyperparameters. To train and evaluate the proposed model, relevant datasets from experimental tests on various rocks were retrieved and GS and K-fold cross-validation methods were adapted to eliminate the overfitting or underfitting problem of the SVR model. From the analysis results, it is found that the hybrid GS-SVR model has higher prediction accuracy and less error compared with the original SVR model and existing analytical models. In addition, a sensitivity analysis was performed to examine the relative importance score of the three input variables (three-dimensional roughness, normal stress, and basic friction angle). The normal stress has the greatest effect on the peak dilation angle, followed by the basic friction angle and the least three-dimensional roughness.

## Figures and Tables

**Table 1:** An overview of existing shear strength models.

References	Shear Strength Model	Peak Dilation Angle
[41]	$\tau_{p}=\sigma_{n}tan\left[\phi_{b}+JRC\cdot {log}_{10}\left(\frac{JCS}{\sigma_{n}}\right)\right]$	$i_{p}=JRC\cdot {log}_{10}\left(\frac{JCS}{\sigma_{n}}\right)$
[37]	$\tau_{p}=\sigma_{n}tan\left[\phi_{b}+i_{0}e^{-k_{1}\sigma_{n}}\right]$	$i_{p}=i_{0}e^{-k_{1}\sigma_{n}}$
[42]	$\tau_{p}=\sigma_{n}tan\left[\phi_{b}+i_{0}{\left(1-\frac{\sigma_{n}}{\sigma_{c}}\right)}^{k_{2}}\right]$	$i_{p}=i_{0}{\left(1-\frac{\sigma_{n}}{\sigma_{c}}\right)}^{k_{2}}$
[43]	$\tau_{p}=\sigma_{n}tan\left\{\phi_{b}+a{\left(SRP\right)}^{c}{\left[{log}_{10}\left(\frac{JCS}{\sigma_{n}}\right)\right]}^{d}+I\right\}$	$i_{p}=a{\left(SRP\right)}^{c}{\left[{log}_{10}\left(\frac{JCS}{\sigma_{n}}\right)\right]}^{d}+I$
[44]	$\tau_{p}=\sigma_{n}tan\left(\phi_{b}+i_{0}\frac{{\left(\sigma_{c}/\sigma_{n}\right)}^{p}}{q+{\left(\sigma_{c}/\sigma_{n}\right)}^{p}}\right)$	$i_{p}=i_{0}\frac{{\left(\sigma_{c}/\sigma_{n}\right)}^{p}}{q+{\left(\sigma_{c}/\sigma_{n}\right)}^{p}}$
[45]	$\tau_{p}=\sigma_{n}tan\left(\phi_{b}+p\frac{\left(\sigma_{t}/\sigma_{n}\right)}{1+\left(\sigma_{t}/\sigma_{n}\right)}\right)$	$i_{p}=p\frac{\left(\sigma_{t}/\sigma_{n}\right)}{1+\left(\sigma_{t}/\sigma_{n}\right)}$
[38]	$\tau_{p}=\sigma_{n}tan\left[\phi_{b}+\frac{4A_{0}\theta_{max}^{}}{C+1}\cdot \left(1+e^{-\frac{1}{9A_{0}}\cdot \frac{\theta_{max}^{}}{C+1}\cdot \frac{\sigma_{n}}{\sigma_{t}}}\right)\right]$	$i_{p}=\frac{4A_{0}\theta_{max}^{*}}{C+1}\cdot \left(1+e^{-\frac{1}{9A_{0}}\cdot \frac{\theta_{max}^{*}}{C+1}\cdot \frac{\sigma_{n}}{\sigma_{t}}}\right)$
[46]	$\tau_{p}=\sigma_{n}tan\left[\phi_{b}+\frac{10A_{0}\theta_{max}^{*}}{1+C}\cdot \frac{\sigma_{t}/\sigma_{n}}{1+\left(\sigma_{t}/\sigma_{n}\right)}\right]$	$i_{p}=\frac{10A_{0}\theta_{max}^{*}}{1+C}\cdot \frac{\sigma_{t}/\sigma_{n}}{1+\left(\sigma_{t}/\sigma_{n}\right)}$
[47]	$\tau_{p}=\sigma_{n}tan\left[\begin{array}{l} \phi_{b}+\theta_{A}e^{-{\left(\theta_{max}^{*}\right)}^{0.89}\left(\frac{\sigma_{n}}{\sigma_{c}}\right)}+\\ {\left(\theta_{max}^{*}\right)}^{1.07}{\left(\frac{\sigma_{n}}{\sigma_{c}}\right)}^{0.42\ln\left[{\left(\theta_{max}^{*}\right)}^{1.07}\right]-1.33} \end{array}\right]$	$\begin{array}{l} i_{p}=\theta_{A}e^{-{\left(\theta_{max}\right)}^{0.89}\left(\frac{\sigma_{n}}{\sigma_{c}}\right)}+\\ {\left(\theta_{max}\right)}^{1.07}{\left(\frac{\sigma_{n}}{\sigma_{c}}\right)}^{0.42\ln\left[{\left(\theta_{max}\right)}^{1.07}\right]-1.33} \end{array}$
[39]	$\tau_{p}=\sigma_{n}tan\left[\phi_{b}+\frac{\theta_{max}^{*}}{C^{0.45}}e^{-\frac{\sigma_{n}}{JCS}C^{0.75}}\right]$	$i_{p}=\frac{\theta_{max}^{*}}{C^{0.45}}e^{-\frac{\sigma_{n}}{JCS}C^{0.75}}$
[48]	$\tau_{p}=\sigma_{n}tan\left[\varphi_{b}+JRC{log}_{10}\left(\frac{JCS}{\sigma_{n}}\right)+{\left[\bar{\theta_{p}}\right]}_{x}\right]$	$i_{p}=JRC{log}_{10}\left(\frac{JCS}{\sigma_{n}}\right)+{\left[\bar{\theta_{p}}\right]}_{x}$
[40]	/	$\begin{array}{l} i_{p}=\theta_{max}^{*}\left[1-10^{\frac{log\left(\frac{\sigma_{n}}{\sigma_{c}}\right)-log\left(A_{0}\right)}{C}}\right]+\\ \frac{\theta_{max}^{*}}{1+C}\left(10^{\frac{log\left(\frac{\sigma_{n}}{\sigma_{c}}\right)-log\left(A_{0}\right)}{C}}\right) \end{array}$
[22]	/	$i_{p}=\frac{\theta_{max}^{*}}{1+C}+\frac{\theta_{cr1}^{*}C}{1+C}-\frac{{\left(\theta_{max}^{*}-\theta_{cr1}^{*}\right)}^{C+1}}{\left(1+C\right)\left(\theta_{max}^{*}-\theta_{cr2}^{*}\right)}$

**Table 2:** Statistical description of inputs and output.

Variables	Type	Maximum	Minimum	Mean	Standard Deviation	Kurtosis	Skewness
*A*_0_	Input	0.69	0.43	0.51517	0.05727	2.76052	1.54207
*C*	Input	13	3.21	8.12258	2.18752	−0.36112	−0.43657
θ_max_^*^ (°)	Input	90	39	68.11685	12.15342	−0.65228	−0.30474
*σ_c_* (MPa)	Input	173	10	64.52247	54.23245	−0.33911	1.08714
*σ_n_* (MPa)	Input	8	0.2	1.95169	1.48853	4.13201	1.80875
*φ_b_* (°)	Input	37	28	33.53933	2.64169	−0.13365	−0.66941
*i_p_* (°)	Output	39.3	8.5	24.89888	6.32154	−0.02348	−0.1438

**Table 3:** Performance comparison of the proposed GS-SVR and SVR models.

Model	Training Set				Test Set			
	R^2^	Adj. R^2^	RMSE	MAPE	R^2^	Adj. R^2^	RMSE	MAPE
GS-SVR	0.959	0.959	1.138	3.1%	0.891	0.884	1.798	10.8%
SVR	0.868	0.866	2.102	7.8%	0.780	0.767	3.237	12.7%

**Table 4:** Comparison between the measured peak dilation angle and the calculated values by different models.

Rock Type	Peak Dilation Angle (°)							
	Measured	GS-SVR Model	Ban (2023) Model [22]	Ban (2020) Model [40]	Xia (2014) Model [38]	Yang (2016) Model [39]	Grasselli (2001) Model [79]	Tatone (2010) Model [80]
Sandstone [80]	36.4	32.63	32.4	33.2	33	24.5	33.8	33.6
	32.1	32.39	31.7	31.7	30.2	24.6	31.2	31.6
	31.1	30.82	28.9	29.1	27.3	23.3	28.8	29.2
	30.3	27.30	30.4	30.3	27.3	24.8	27.6	28.6
	28.5	25.85	29	29.3	25.7	24.4	26.1	27.1
	27.6	29.01	27.2	27.1	23.1	22.9	24.2	25
Sandstone [38]	24.6	18.32	19.7	19.7	17.3	18.4	22	22.4
	15	15.75	17	17	15.1	16.6	18.1	18.8
	14.7	14.41	15.4	15.4	13.6	14.9	15.1	15.8
	13.3	13.38	14.2	14.2	12.6	13.4	12.8	13.6
	8.5	8.44	12.4	12.4	11.4	10.9	10.1	10.7
	31.2	28.29	28.5	28.6	24.5	24.9	24.5	25.1
	25.3	25.0	24.9	24.9	20.7	22.9	19.9	20.8
	20.7	21.80	22.6	22.6	18.5	21	16.9	17.8
	19.3	18.85	20.9	20.9	17.2	19.2	15.1	15.8
	13.1	14.09	18.4	18.4	16.1	16.2	13.5	13.9
	39.3	35.78	31.2	31.4	36.9	25.6	26.5	27
	32.6	32.31	27.7	27.8	31.9	23.6	22.6	23.4
	27.6	28.81	25.5	25.5	28.6	21.7	19.6	20.5
	25.4	20.54	23.8	23.8	26.4	20	17.5	18.4
	19.5	19.78	21.3	21.3	24.1	17	15.2	15.8
Sandstone [39]	37.1	33.40	28.3	38.9	29	29	29	29
	26.2	26.48	28.2	39.9	38.6	31.5	29	29
	29	29.29	29.9	34.5	29	29	25.2	25.2
	29	29.96	28.6	31.2	22.9	27.6	22.9	22.9
	24.3	24.59	27.1	28.6	24.3	26.8	21.5	19.9
	29	22.47	28.8	31.8	27.2	28.1	21.7	20.5
	27.4	25.95	26.2	27.5	19.7	25.8	18.6	17.3
	26.2	25.91	25.5	26.7	18.1	25.4	17	17
	24.4	24.69	23.7	24.5	15.8	24.4	15.8	14.7
	27.3	21.03	25.8	27.3	19.9	25.6	17.5	16.6
Marble [79]	25.9	26.06	27.5	30.2	27.2	25.9	27.2	27.2
	16.1	16.38	20.3	20.3	20.4	17.2	24.6	24.6
	17.1	19.32	23.5	23.9	19.2	21.2	25.9	25.9
	19.9	20.19	20.6	20.9	21.2	19.5	22.1	22.5
	22.5	22.20	20.4	20.6	21.9	18.6	23.6	23.6
	21.9	20.92	20.3	20.6	17.9	20	22.5	23
	19	19.67	19.2	19.4	19	20.4	21.2	22.1
	22.1	21.80	19.2	19.4	17.7	19.6	20.9	21.7
	23	22.54	17.9	18	17.9	17.2	22.5	22.5
	22.9	22.61	24	24.4	22.9	21.2	25.9	25.9
	22.2	21.91	22.8	23.1	19.5	20.4	26.6	25.3
	17	17.27	19.8	20.3	20.5	17	14.9	17
	10.5	10.78	8.4	8.4	14	4.9	12.3	11.1
	10.8	10.41	13.8	14	16.3	9.5	29.1	13.1
Granite [79]	34.1	33.81	35	42.3	33	35.7	29.5	30.4
	33.7	33.99	34.3	44.5	36.5	35.7	31.8	32.2
	31.5	30.10	34.2	36.9	31.5	29	29	29.6
	31	30.71	33.9	35.4	29.1	25.5	30.1	30.1
	34.9	31.36	34.6	42.6	33.5	32.7	31	31.9
	34.2	34.49	34.8	42	32.7	31.9	31	31.9
	35.6	35.31	33	44.6	38.8	32.7	32.7	33.5
Granite [39]	27.9	27.63	27.6	34.2	33.2	28	28	28
	28.7	28.98	27.9	32.2	28	28	27.1	27.1
	29.4	28.67	27.1	29.7	23.8	27.4	25.1	25.1
	27.1	27.39	27	29.8	24.9	27.1	24.4	24.4
	24	24.18	23.8	24.8	19.5	24	21.4	21.4
	22.7	22.99	23.1	23.9	19.2	23.4	20.4	20.4
	22.6	20.57	22.2	22.8	20	22	19.6	20
	25.8	25.52	24.8	26.4	23.4	24.4	20	20.3
	23.3	23.01	23.4	24.5	19.7	23.3	18.3	18.6
	23.8	24.09	25.1	27.1	22.3	24.8	19	19
Limestone [79]	28.1	28.39	26.9	30.5	27	28.1	24.6	25.9
	27	27.28	28	35.4	28.1	31.7	28.1	28.1
	20	19.70	19.7	20.4	19	17.9	20	19
	26	23.93	24.8	27.1	24.9	23.2	23.2	22.5
	22.1	22.39	23.8	26.2	24.1	23.1	23.1	22.7
	28.1	28.39	27.9	33.4	33.3	28.1	28.1	28.1
	21.6	21.30	23.5	25.5	25.8	21.6	21.6	21.6
Cement mortar [40]	33.6	30.27	24.6	24.7	25.7	21.7	24.7	25.4
	25.2	26.73	21.5	21.5	23.1	20	20.9	21.5
	22.5	23.33	19.5	19.5	21.3	18.4	17.7	18.5
	20.5	20.22	18	18	20	17	15.5	16.3
	30.3	25.53	21.5	21.5	22.9	19.6	23.6	24
	22.5	21.53	18.6	18.6	20.7	17.7	20	20.7
	21.2	21.48	16.8	16.8	19.1	16.3	16.9	17.7
	19.4	19.10	15.5	15.5	18.1	15	14.7	15.5
	32.1	27.02	23.8	23.9	27.9	22.1	24.7	25
	23.8	24.31	20.7	20.8	25.4	20	21.3	21.9
	21.8	20.09	18.8	18.8	23.5	18.1	18.4	19.3
	19.9	19.62	17.4	17.4	22.1	16.3	16.3	17.1
	26.3	26.01	22.7	22.7	27.9	20.5	24.3	24.7
	20.5	22.88	19.8	19.8	25.4	18.7	21.3	21.9
	19.7	19.99	18	18	23.6	16.9	18.5	19.3
	18.2	17.47	16.6	16.7	22.3	15.4	16.4	17.3
	36.2	33.91	27.9	28.1	33.2	23.3	26.7	27
	27.6	25.92	24.7	24.8	29.8	21.5	22.9	23.6
	23.1	25.19	22.6	22.7	27.3	20	19.9	20.7
	21.5	21.21	21	21.1	25.5	18.6	17.7	18.5

## Data Availability

Data available on request due to privacy restrictions.

## Supplementary Materials

The following supporting information can be downloaded at: https://www.mdpi.com/article/10.3390/ma16196382/s1.

## Abbreviations

*τ_p_*	Shear strength
*i_p_*	Peak dilation angle
*i*_0_	Dilation angle under zero normal stress (°)
*φ_b_*	Basic friction angle
*k*_1_ *k*_2_	Fitting constant
SRP	Stationary roughness profile
*p*, *q*	Regression coefficients
*C*	Roughness parameter characterizing distribution of apparent dip angles over joint surface
*θ_A_*	Average angle of asperities facing shear direction
JRC	Joint roughness coefficient
JCS	Joint wall compressive strength
*σ_n_*	Normal stress
*σ_c_*	Uniaxial compressive strength
*a*, *c*, *d*	Fitting constants
*σ_t_*	Tension strength
*A*_0_	Maximum potential contact area for the specified shear direction
*θ*_*max*_	Maximum apparent dip angle (°)
${\left[\bar{\theta_{p}}\right]}_{x}$	Average of the mean profile angles
$\theta_{cr2}^{*}$	Fitting constant
